# Acetylation of CspC Controls the Las Quorum-Sensing System through Translational Regulation of *rsaL* in Pseudomonas aeruginosa

**DOI:** 10.1128/mbio.00547-22

**Published:** 2022-04-25

**Authors:** Shouyi Li, Xuetao Gong, Liwen Yin, Xiaolei Pan, Yongxin Jin, Fang Bai, Zhihui Cheng, Un-Hwan Ha, Weihui Wu

**Affiliations:** a State Key Laboratory of Medicinal Chemical Biology, Key Laboratory of Molecular Microbiology and Technology of the Ministry of Education, Department of Microbiology, College of Life Sciences, Nankai University, Tianjin, China; b Department of Biotechnology and Bioinformatics, Korea University, Sejong, Republic of Korea; Laboratory of Microbiology Signals and Microenvironment LMSM EA 4312; Harvard Medical School

**Keywords:** acetylation, CspC, itaconate, *Pseudomonas aeruginosa*, quorum sensing

## Abstract

Pseudomonas aeruginosa is a ubiquitous pathogenic bacterium that can adapt to a variety environments. The ability to effectively sense and respond to host local nutrients is critical for the infection of P. aeruginosa. However, the mechanisms employed by the bacterium to respond to nutrients remain to be explored. CspA family proteins are RNA binding proteins that are involved in gene regulation. We previously demonstrated that the P. aeruginosa CspA family protein CspC regulates the type III secretion system in response to temperature shift. In this study, we found that CspC regulates the quorum-sensing (QS) systems by repressing the translation of a QS negative regulatory gene, *rsaL*. Through RNA immunoprecipitation coupled with real-time quantitative reverse transcription-PCR (RIP-qRT-PCR) and electrophoretic mobility shift assays (EMSAs), we found that CspC binds to the 5′ untranslated region of the *rsaL* mRNA. Unlike glucose, itaconate (a metabolite generated by macrophages during infection) reduces the acetylation of CspC, which increases the affinity between CspC and the *rsaL* mRNA, leading to upregulation of the QS systems. Our results revealed a novel regulatory mechanism of the QS systems in response to a host-generated metabolite.

## INTRODUCTION

Pseudomonas aeruginosa is an opportunistic pathogen that predominates as a major cause of pulmonary infections in patients with cystic fibrosis and chronic obstructive pulmonary disease (COPD) (1, 2). The successful colonization of P. aeruginosa depends on its ability to evade host immune clearance and efficiently acquire and utilize available nutrients by orchestrating global gene expression in response to the host environment.

P. aeruginosa possesses a variety of mechanisms to sense and respond to host signals, such as two-component regulatory systems and extracytoplasmic function (ECF) sigma factors (3–6). Surface structural proteins are also involved in signal sensing. For instance, the type IV pilus component PilY1 is involved in surface sensing and regulates synthesis of the secondary messenger c-di-GMP and expression of various virulence factors (7–9). The outer membrane protein OprF directly binds to interferon gamma (IFN-γ) and activates the production of the virulence determinant PA-I lectin through the quorum-sensing (QS) system (10).

Three central QS systems have been discovered in P. aeruginosa, namely, the Las, Rhl, and PQS systems, which control biofilm formation, bacterial stress responses, and the production of various virulence factors, including hemolysins, alkaline protease, exotoxin-A, elastases, siderophores, hydrogen cyanide, pyocyanin, rhamnolipids, etc. (11, 12). The signal molecules of the Las and Rhl QS systems are *N*-(3-oxododecanoyl)-homoserine lactone (3-oxo-C12-HSL) and *N*-butyrylhomoserine lactone (C4-HSL), which are synthesized by LasI and RhlI and recognized by LasR and RhlR, respectively (13, 14). For the PQS system, there are two signal molecules, namely, 2-heptyl-3-hydroxy-4-quinolone (PQS) and 2-heptyl-4-quinolone (HHQ), both of which are recognized by MvfR (PqsR) (15, 16).

The three QS systems are interconnected hierarchically. At the top of the regulatory hierarchy is the Las system (17, 18). After binding to 3-oxo-C12-HSL, LasR activates the transcription of *lasI*, *rhlI*, *rhlR*, *pqsR*, and genes involved in the synthesis of HHQ and PQS (19–22). In addition, LasR directly induces the expression of *rsaL*, which encodes a DNA binding protein (23). By binding to the promoter region of *lasI*, RsaL represses the transcription of *lasI*, thus forming a negative feedback loop (23–25).

Besides the endogenous signal molecules, the P. aeruginosa QS systems are influenced by a variety of nutritional conditions (12). For instance, phosphate depletion activates the QS systems through the two-component regulatory system PhoR-PhoB (26). van Delden et al. demonstrated that amino acid starvation triggers the stringent response, which induces the Las and Rhl systems (27). In P. aeruginosa, Crc is a global regulator that controls catabolite repression. Mutation in the *crc* gene resulted in downregulation of the *rhlI* gene (28, 29), indicating a possible interrelationship between carbon metabolism and the QS systems.

CspA family proteins are small RNA binding proteins that are involved in bacterial response to environmental changes (30–32). P. aeruginosa harbors five CspA family proteins, namely, CspC (PA0456), PA0961, PA1159, CspD (PA2622), and CapB (PA3266) (33). Previously, we found that CspC regulates the translation of the type III secretion system (T3SS) master regulatory gene *exsA* by binding to the 5′ untranslated region (5′UTR) of its mRNA (33). We further demonstrated that the binding affinity of CspC to the *exsA* mRNA is affected by the acetylation at the K41 residue in response to temperature shift, indicating a role of CspC in bacterial response to environmental signals (33). In this study, we explored the roles of the P. aeruginosa CspA family proteins in response to nutrient availability and abundance and found that CspC regulates pyocyanin production without affecting bacterial growth in complete medium. We further found that CspC regulates the QS systems by binding to the 5′UTR of the *rsaL* mRNA. In addition, we demonstrated that carbon sources affect the acetylation level of CspC, which affects its regulatory effect on *rsaL*. Overall, our results reveal a novel regulatory mechanism of the QS systems in response to environmental nutrients.

## RESULTS

### CspC is required for pyocyanin production.

**FIG 1:**
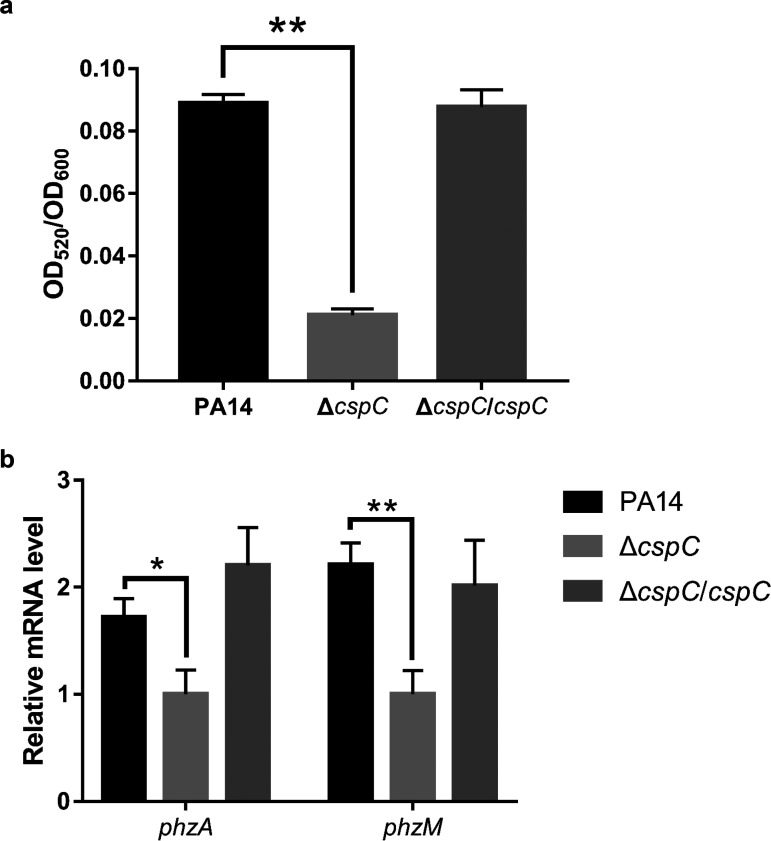
CspC is required for pyocyanin production. Indicated strains were grown overnight in LB at 37°C. (a) Pyocyanin levels in the supernatants of the overnight bacterial cultures. (b) mRNA levels of the pyocyanin synthesis genes, including overall *phzA* (*phzA1* and *phzA2*) and *phzM*, were determined by qRT-PCR. Data represent the means from three independent experiments, and error bars indicate standard deviations. **, *P < *0.01; *, *P < *0.05 by Student's *t* test.

To examine whether the CspA family proteins are involved in bacterial response to cell density and extracellular nutrients, we monitored the growth curves of the strains with mutations in each of the *cspA* family genes. All of the mutants grew normally in LB medium (see Fig. S1a in the supplemental material). However, the color of the Δ*cspC* mutant culture remained yellow at an optical density at 600 nm (OD_600_) of 3.0, while the others’ turned blue-green, indicating a defect in pyocyanin production (Fig. S1b). Indeed, mutation of the *cspC* gene reduced the extracellular amount of pyocyanin (Fig. 1a and b). In PA14, genes *phzM* and *phzS* and two nearly identical operons, *phzA1*-*G1* and *phzA2*-*G2*, are involved in pyocyanin synthesis (34). We determined the mRNA levels of *phzM* and overall *phzA* (*phzA1* plus *phzA2*) by using quantitative reverse transcription-PCR (qRT-PCR) primers targeting *phzM* and the common sequences of *phzA1* and *phzA2* (Table S1). Mutation of *cspC* resulted in downregulation of the overall *phzA* and *phzM* (Fig. 1b). Complementation with a *cspC* gene restored the expression of the *phz* genes and production of pyocyanin (Fig. 1a). These results reveal a role of CspC in regulating the production of pyocyanin.

10.1128/mbio.00547-22.1 FIG S1 (a) Growth curves of the indicated strains. The growth of the bacteria was monitored by measuring the OD_600_ every hour for 12 h. (b) Pyocyanin levels in the supernatants of the overnight bacterial cultures. ***, *P* < 0.001 by Student’s *t* test. Download FIG S1, TIF file, 0.2 MB. Copyright © 2022 Li et al. 2022 Li et al. https://creativecommons.org/licenses/by/4.0/ This content is distributed under the terms of the Creative Commons Attribution 4.0 International license.

10.1128/mbio.00547-22.2 TABLE S1 Bacterial strains, plasmids, and primers used in this study. Download Table S1, RTF file, 0.3 MB. Copyright © 2022 Li et al. 2022 Li et al. https://creativecommons.org/licenses/by/4.0/ This content is distributed under the terms of the Creative Commons Attribution 4.0 International license.

### CspC controls the production of QS signal molecules.

**FIG 2:**
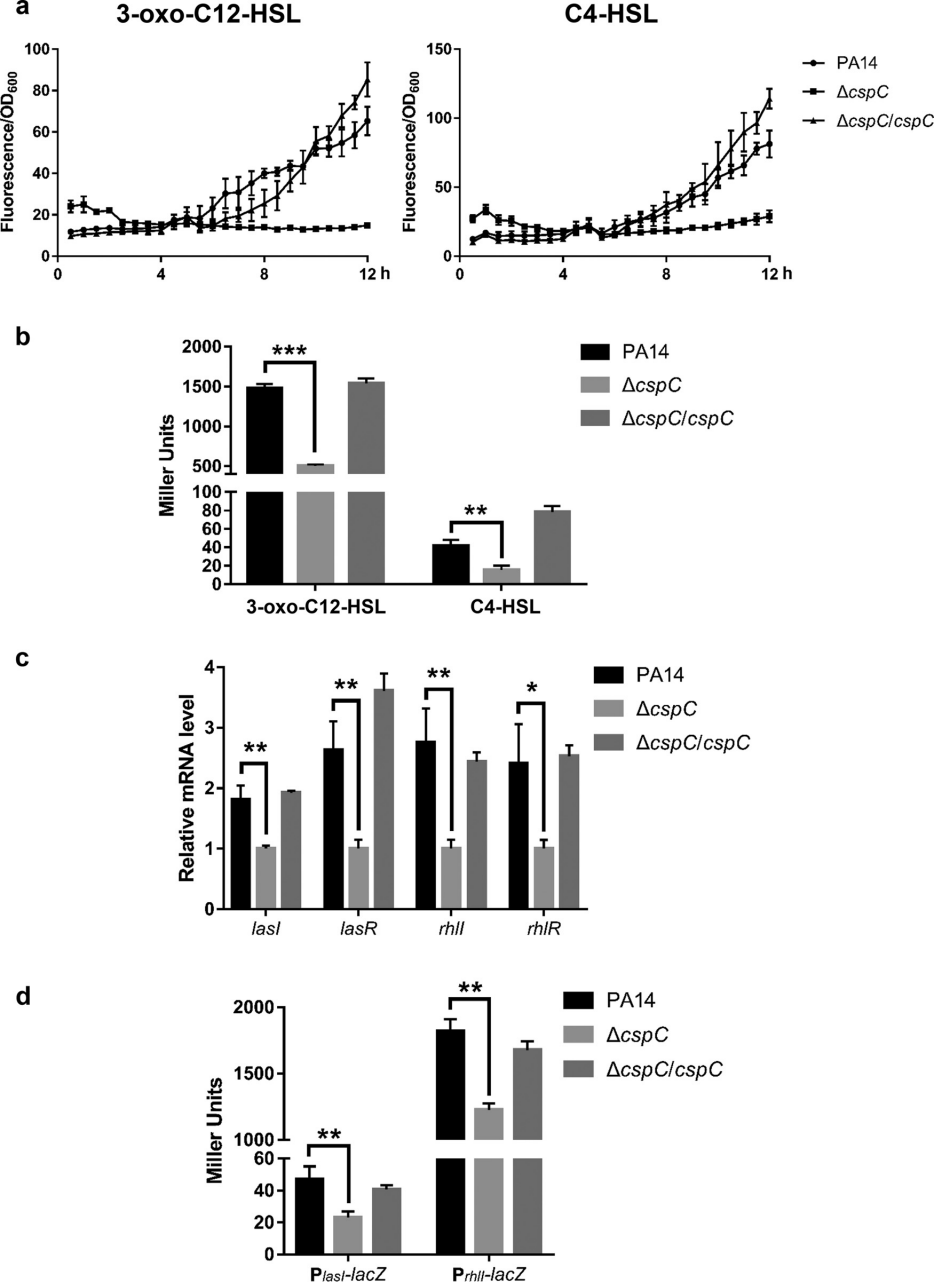
CspC controls the production of QS signal molecules. (a) The relative levels of 3-oxo-C12-HSL and C4-HSL of indicated strains. The indicated strains were grown overnight. The bacterial supernatants were mixed with the reporter strains at a volume ratio of 1:1. The GFP fluorescence and OD_600_ were measured every 30 min at 37°C for 12 h. The data were calculated as GFP fluorescence/OD_600_. (b) 3-Oxo-C12-HSL and C4-HSL contents of indicated strains measured with E. coli reporter strains in LB. Miller units are used for the mean results from at least three independent experiments. ***, *P < *0.001; **, *P < *0.01 by Student's *t* test. (c) Relative mRNA levels of *lasI*, *lasR*, *rhlI*, and *rhlR.* Total RNA of indicated strains was isolated from bacteria grown overnight, and mRNA levels of *lasI*, *lasR*, *rhlI*, and *rhlR* were determined by qRT-PCR. Data represent the means from three independent experiments, and error bars indicate standard deviations. **, *P < *0.01; *, *P < *0.05 by Student's *t* test. (d) Bacteria carrying P*_lasI_*-*lacZ* and P*_rhlI_*-*lacZ* were grown in LB to an OD_600_ of 0.3. Miller units are used for mean results from three independent experiments. **, *P < *0.01 by Student's *t* test.

The synthesis of pyocyanin is regulated by the quorum-sensing systems. To understand the mechanism of CspC-mediated regulation on pyocyanin synthesis, we examined extracellular amounts of the QS signal molecules 3-oxo-C12-HSL and C4-HSL by utilizing P. aeruginosa reporter strains (35). Compared to wild-type PA14 and the complemented strain, the Δ*cspC* mutant produced smaller amounts of 3-oxo-C12-HSL and C4-HSL (Fig. 2a). The results were further confirmed by utilizing Escherichia coli-based reporter systems (Fig. 2b). In agreement with defective production of the QS signals, the mRNA levels of *lasI*, *lasR*, *rhlI*, and *rhlR* as well as the promoter activities of *lasI* and *rhlI* were decreased in the Δ*cspC* mutant and restored to wild-type levels by complementation with the *cspC* gene (Fig. 2c and d).

### CspC controls the QS systems through *rsaL*.

**FIG 3:**
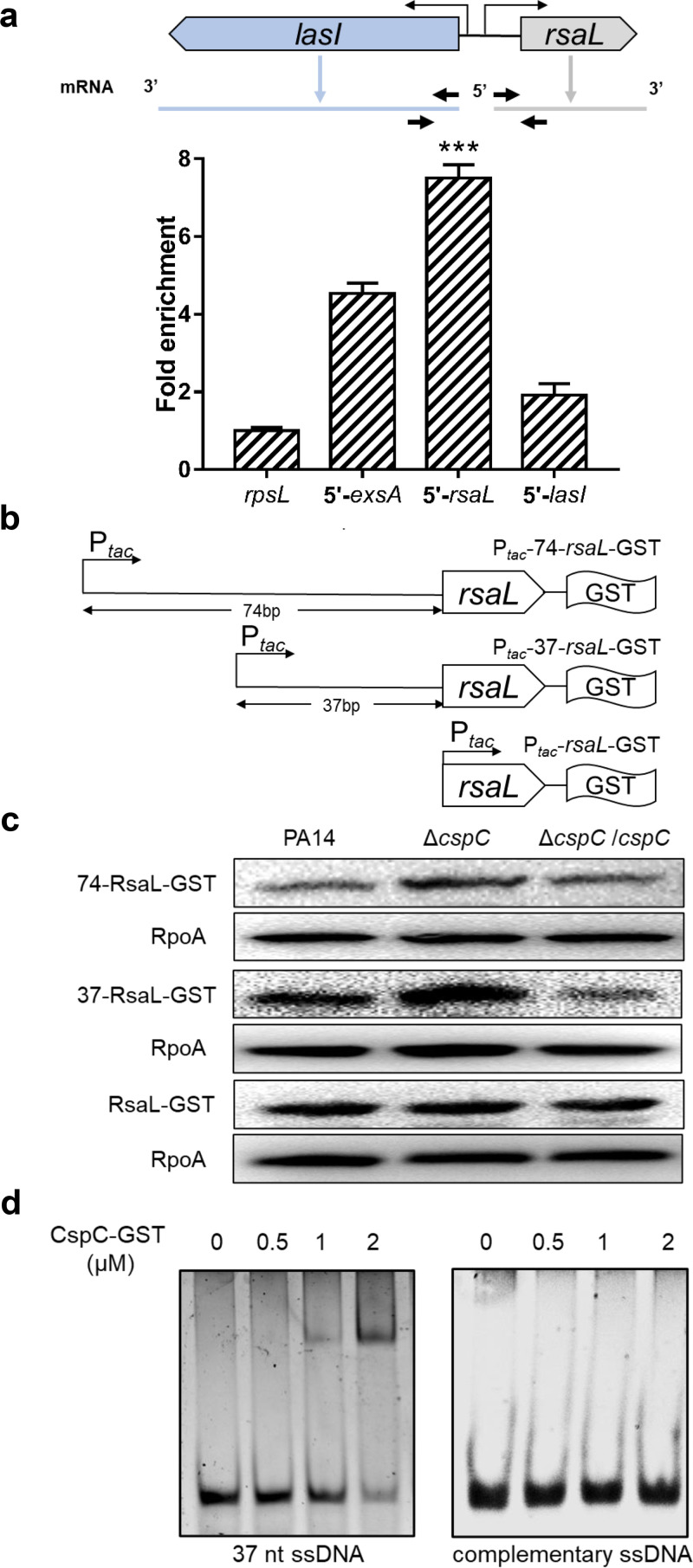
CspC binds to the 5′UTR of the *rsaL* mRNA. (a) Fold enrichment of the indicated fragments tested by an RIP-coupled qRT-PCR assay. Locations of the primers used in the qRT-PCR are indicated by arrows. The *exsA* gene was used as a positive control, and the *rpsL* gene was used as an internal control. Data represent the means from three independent experiments, and error bars indicate standard deviations. ***, *P < *0.001 compared to the other samples by Student's *t* test. (b) Schematic diagram of the *rsaL*-GST fusions driven by a *tac* promoter with indicated length of upstream regions. (c) Bacteria carrying the *rsaL*-GST with indicated upstream segments (P*_tac_*-74/37/-*rsaL*-GST) were grown in LB containing 150 μg/mL carbenicillin. The RsaL-GST and RpoA levels were determined by Western blotting. (d) The purified CspC-GST was incubated with a 37-nt ssDNA that represents the 37-nt 5′-UTR of the *rsaL* mRNA and a complementary ssDNA for 30 min at 25°C. The samples were subjected to electrophoresis in a native gel, followed by staining with SYBR Gold nucleic acid gel stain.

Previously, we demonstrated CpsC regulates gene expression by binding to mRNA (33). To elucidate the mechanism of CspC-mediated regulation on the QS systems, we checked our previous RNA immunoprecipitation (RIP) sequencing result and found enrichment of the region upstream of the *rsaL* gene by CspC (33). By performing a RIP-coupled qRT-PCR assay, we verified the binding of CspC to the 5′UTR of the *rsaL* mRNA (Fig. 3a), whereas the neighboring 5′UTR of the *lasI* mRNA was not enriched by CspC (Fig. 3a), indicating possible posttranscriptional regulation of *rsaL* by CspC.

To examine whether CspC controls the translation of the *rsaL* mRNA, we constructed a C-terminal *gst*-tagged *rsaL* driven by a P*_tac_* promoter. It has been demonstrated that the transcription of the *rsaL* mRNA initiates at 74 nucleotides (nt) upstream of its start codon (23). Thus, we included the 74-bp region in the construct, resulting in P*_tac_*-74-*rsaL*-*gst* (Fig. 3b). The RsaL–glutathione-*S*-transferase (GST) level was higher in the Δ*cspC* mutant (Fig. 3c). To narrow down the region that is involved in the translational regulation, we reduced the 5′UTR to 37 nt (designated P*_tac_*-37-*rsaL*-*gst*), which also resulted in a higher level of RsaL-GST in the Δ*cspC* mutant (Fig. 3c). However, replacement of the native 5′UTR sequence with an exogenous ribosome binding sequence (designated P*_tac_*-*rsaL*-*gst*) resulted in similar levels of RsaL-GST in wild-type PA14 and the Δ*cspC* mutant (Fig. 3c), indicating an essential role of the 37-nt 5′UTR in the CspC-mediated translational regulation of *rsaL*.

We then examined whether CspC directly binds to the 37-nt 5′UTR by electrophoretic mobility shift assay (EMSA). It has been demonstrated that the CspA family proteins bind to single-stranded DNA (ssDNA) as efficiently as RNA (36, 37). Thus, we used ssDNAs in the EMSAs. The 37-nt ssDNA that represents the *rsaL* 5′UTR was bound by CspC, whereas no obvious binding was observed between the complementary ssDNA and CspC (Fig. 3d). In combination, these results demonstrate that CspC represses the translation of the *rsaL* mRNA by binding to its 37-nt 5′UTR.

**FIG 4:**
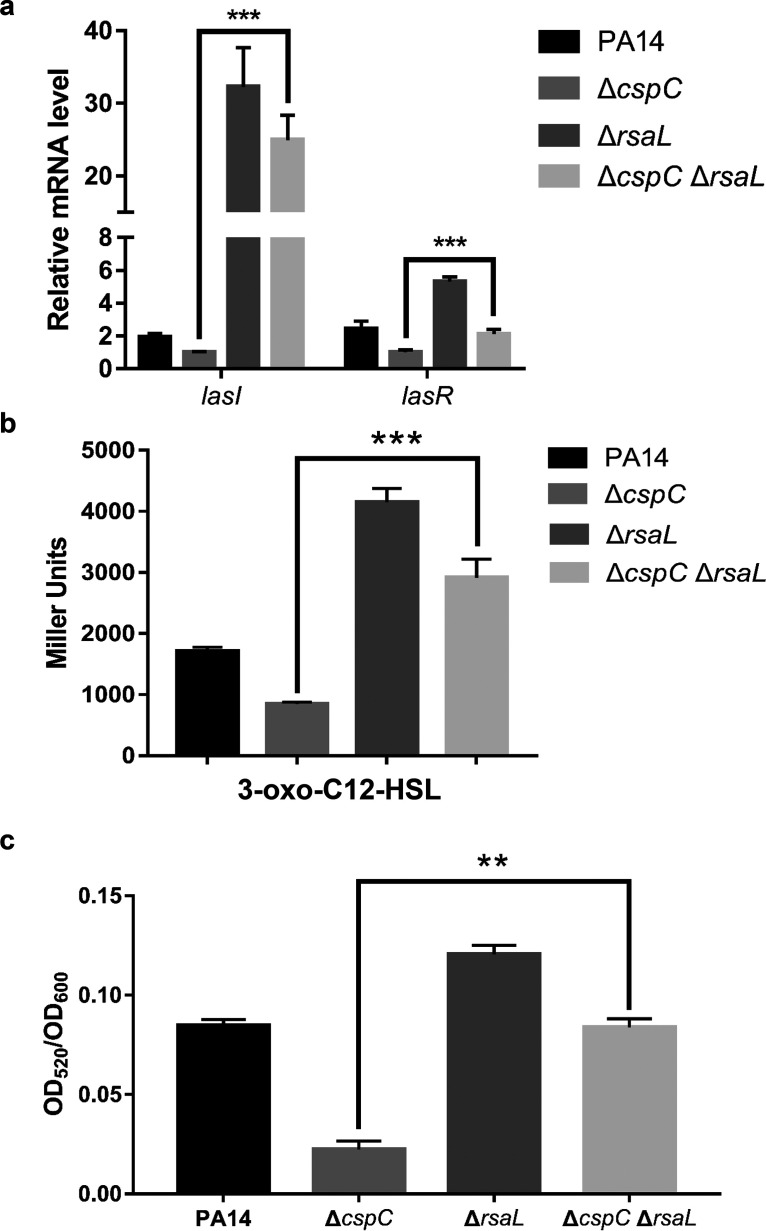
CspC controls the QS systems through *rsaL*. The bacteria were grown overnight in LB at 37°C. (a) The relative mRNA levels of *lasI* and *lasR* were determined by qRT-PCR. Data represent the mean from three independent experiments, and error bars indicate standard deviations. (b) The relative 3-oxo-C12-HSL levels in the supernatant of the bacterial cultures were measured with the E. coli reporter strain. Miller units are the mean results from three independent experiments. (c) The pyocyanin levels in the supernatant of the bacterial cultures. Data represent the means from three independent experiments, and error bars indicate standard deviations. ***, *P < *0.001; **, *P < *0.01 by Student's *t* test.

Since RsaL represses the transcription of *lasI*, the defective production of pyocyanin by the Δ*cspC* mutant might be due to the derepression of *rsaL*. Indeed, deletion of the *rsaL* gene in the Δ*cspC* mutant increased the expression of the *lasI* and *lasR* genes (Fig. 4a) as well as the production of 3-oxo-C12-HSL and pyocyanin (Fig. 4b and c).

### The K41 residue of CspC is involved in the translational regulation of *rsaL*.

**FIG 5:**
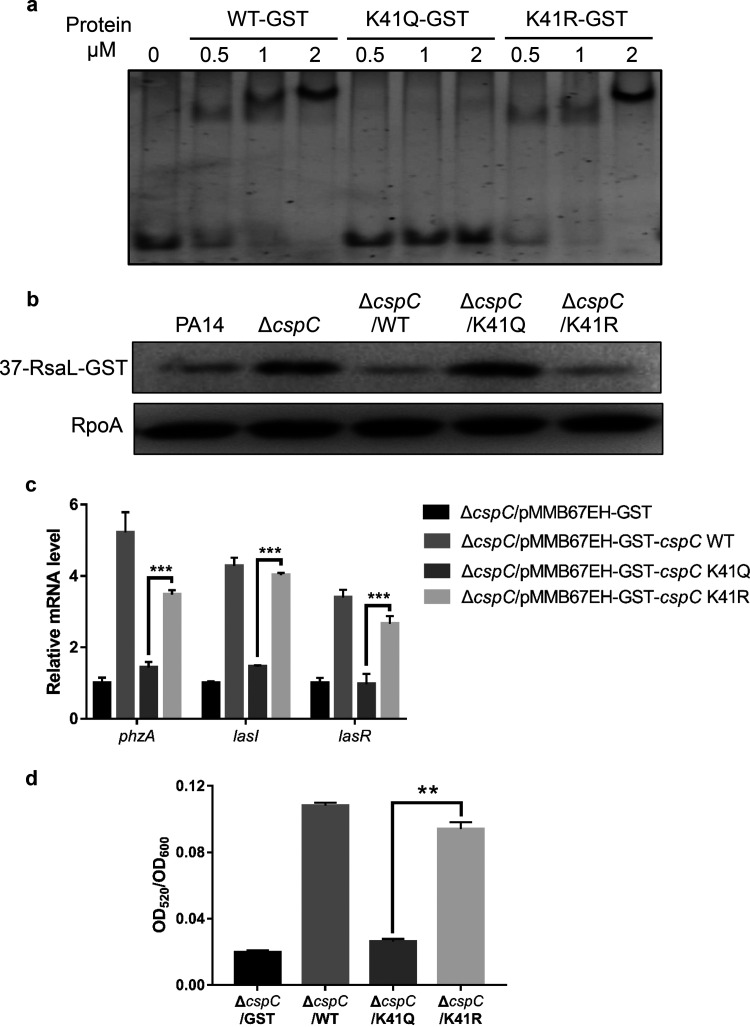
K41 residue of CspC is involved in the translational regulation of *rsaL*. (a) Equal amounts of CspC(WT)-GST, CspC(K41Q)-GST, and CspC(K41R)-GST were incubated with the 37-nt ssDNA for 30 min at 25°C. The samples were subjected to electrophoresis in a native gel, followed by staining with SYBR Gold nucleic acid gel stain. (b) Indicated strains carrying P*_tac_*-37-*rsaL*-GST were grown to an OD_600_ of 1.0 in LB containing 150 μg/mL carbenicillin. The RsaL-GST and RpoA levels were determined by Western blotting. (c and d) Indicated strains were grown overnight in LB containing 150 μg/mL carbenicillin and 1 mM IPTG. (c) The mRNA levels of overall *phzA*, *lasI*, and *lasR* were determined by qRT-PCR. (d) The pyocyanin levels in the supernatants. Data represent the means from three independent experiments, and error bars indicate standard deviations. ***, *P < *0.001; **, *P < *0.01 by Student's *t* test.

Previously we demonstrated that acetylation at K41 of CspC reduces its affinity to the *exsA* mRNA, resulting in derepression of the translation (33). To examine whether K41 acetylation is involved in the CspC-mediated regulation of *rsaL*, we performed EMSA with CspC with K41R and K41Q mutations, which mimic the unacetylated and acetylated states, respectively (38). The K41Q mutation significantly reduced the binding affinity between CspC and the 37-nt ssDNA (Fig. 5a). In the Δ*cspC* mutant carrying P*_tac_*-37-*rsaL*-*gst*, overexpression of the K41Q *cspC* mutant did not affect the expression of RsaL-GST, whereas overexpression of the K41R *cspC* mutant repressed the expression (Fig. 5b). In addition, overexpression of K41R *cspC* but not K41Q *cspC* in the Δ*cspC* mutant restored the expression of the *lasI*, *lasR*, and *phzA* genes as well as the production of pyocyanin (Fig. 5c and d). These results indicate a role of K41 acetylation in the CspC-mediated regulation of *rsaL* and the Las QS system.

### Acetylation of CspC modulates the expression of *rsaL* and the Las QS system in response to itaconate.

**FIG 6:**
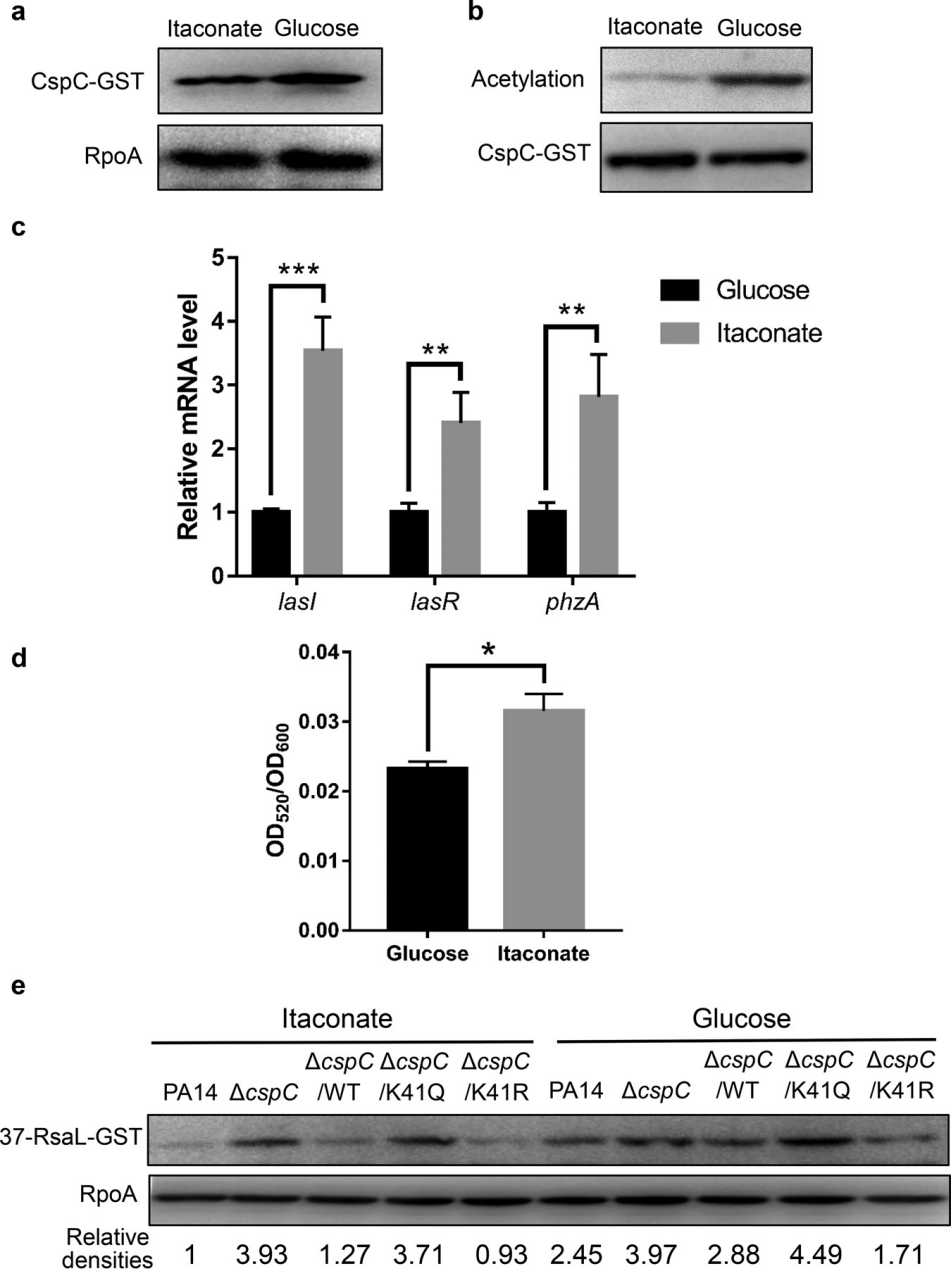
Acetylation of CspC modulates the expression of *rsaL* and the Las QS system in response to itaconate. (a and b) Wild-type PA14 carrying a *cspC*-GST driven by its own promoter was grown at 37°C overnight in M9 medium with glucose or itaconate as the sole carbon source. (a) The amounts of CspC-GST and RpoA were determined by Western blotting. (b) Acetylation and the total amounts of the purified CspC-GST were determined by Western blotting. (c and d) Wild-type PA14 was grown in M9 medium with glucose or itaconate as the sole carbon source at 37°C. (c) The mRNA levels of *lasI*, *lasR*, and overall *phzA* were determined by qRT-PCR. Data represent the means from three independent experiments, and error bars indicate standard deviations. ***, *P < *0.001; **, *P < *0.01; *, *P < *0.05 by Student's *t* test. (d) Pyocyanin levels in the supernatants of the bacterial cultures. (e) Indicated strains carrying P*_tac_*-37-*rsaL*-GST were grown to an OD_600_ of 1.0 in M9 medium with glucose or itaconate as the sole carbon source at 37°C. The RsaL-GST levels were determined by Western blotting. Data represent the results from three independent experiments.

A recent study demonstrated that during lung infection, the host-derived itaconate promotes biofilm formation by P. aeruginosa (39). Gaviard et al. demonstrated alternation of the acetylome of P. aeruginosa by carbon sources (40). Since the QS systems are required for biofilm formation, we suspected that the acetylation of CspC was affected by itaconate, which subsequently regulates the Las QS system through RsaL. Thus, we grew wild-type PA14 in a minimal medium with itaconate or glucose as the sole carbon source. Compared to glucose, itaconate decreased the acetylation of CspC without affecting the expression of CspC (Fig. 6a and b). In addition, itaconate increased the expression of *lasI*, *lasR*, and *phzA* and the production of pyocyanin (Fig. 6c and d).

We then examined the translation of *rsaL* by using P*_tac_*-37-*rsaL*-*gst*. Compared to glucose, itaconate reduced the expression of RsaL-GST in wild-type PA14, whereas deletion of *cspC* resulted in an equally higher level of RsaL-GST in the presence of itaconate and glucose (Fig. 6e). Complementation with K41R *cspC* reduced the expression levels of RsaL-GST in the presence of itaconate and glucose. However, K41Q *cspC* was not able to reduce the expression of RsaL-GST in either of the carbon sources (Fig. 6e). In combination, these results demonstrate that acetylation at K41 modulates the activity of CspC in the regulation of *rsaL* in response to itaconate.

## DISCUSSION

In this study, we demonstrated that the CspA family protein CspC regulates the QS systems by repressing the expression of *rsaL.* The CspC protein directly binds to the 37-nt fragment of the 5′ UTR of the *rsaL* mRNA, which might alter the secondary structure of the mRNA or block the access of ribosome, thus inhibiting the translation.

CspA family proteins are conserved RNA chaperones in bacteria that are involved in bacterial response to environmental signals (41–45). The CspA family proteins usually contain five anti-parallel β-strands and fold into a β-barrel structure (46, 47). In E. coli, CspA and another CspA family protein, CspE, are involved in bacterial cold adaptation by altering mRNA secondary structures, which may affect transcription and mRNA translation (48–50). In Staphylococcus aureus, CspA binds to the 5′UTR of its own mRNA, which disrupts the stem-loop in the 5′UTR and subsequently blocks the processing by RNase III, leading to repression of translation (36). In Salmonella enterica serovar Typhimurium, CspC and CspE play important roles in bacterial response to membrane stress, motility, biofilm formation, and virulence in a mouse systemic infection model (51). One of the targets of CspC and CspE is the *ecnB* mRNA, which encodes a protein involved in bacterial response to starvation. The binding of CspC and CspE to the *ecnB* mRNA protects it from degradation by RNase E (51).

We previously found that in P. aeruginosa, CspC regulates the T3SS by directly binding to the 5′UTR of the *exsA* mRNA, which results in translational repression (33). The binding affinity between CspC and the *exsA* mRNA is reduced by acetylation at K41 of CspC (33). The reversible lysine acetylation is one of the most common posttranslational modifications (PTMs) that play critical roles in bacterial metabolism, response to environmental stresses, and virulence (52–57). For nucleic acid binding proteins, acetylation of lysine residues decreases the positive charge of the protein, thereby affecting the folding of the protein and its affinity to nucleic acids (57). Our previous study demonstrated that the acetylation of CspC is increased when the culture temperature is switched from 25°C to 37°C, indicating a CspC-mediated regulatory mechanism in sensing mammalian host body temperature (33).

Carbon sources play important roles in affecting PTMs in bacteria (58–61). Through proteomic analyses, Gaviard et al. demonstrated that the global protein acetylation of PA14 was altered by different carbon sources (40). When glucose was the sole carbon source, 320 acetylated proteins were identified in PA14, which are mainly involved in carbon metabolism, lipopolysaccharide (LPS) biosynthesis, oxidative stress response, and virulence factor production (62, 63).

**FIG 7:**
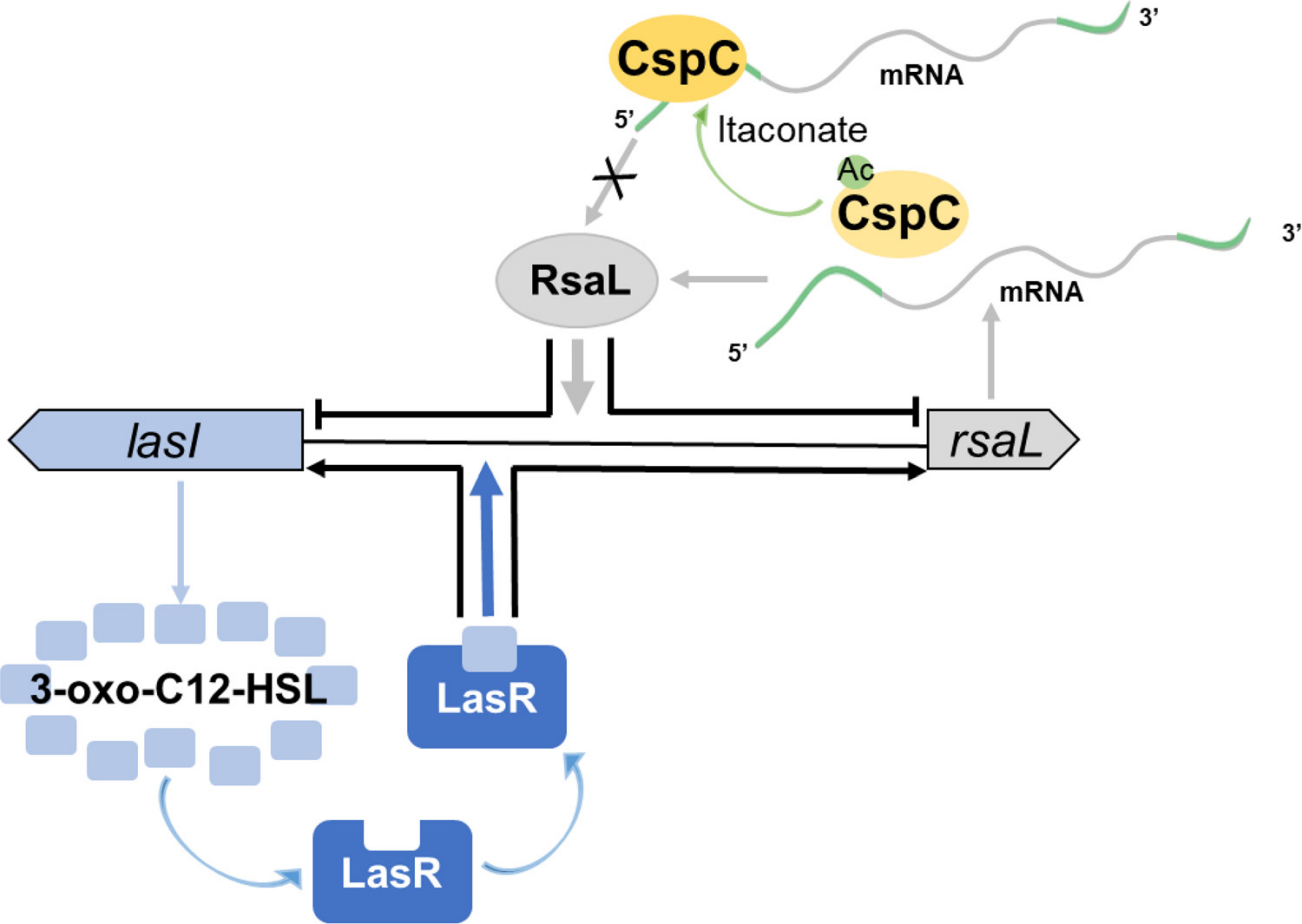
Schematic diagram of the CspC-mediated regulation on *rsaL* and the Las QS system. LasI synthesizes the signal molecule 3-oxo-C12-HSL. After binding to 3-oxo-C12-HSL, LasR binds to the intergenic region between *lasI* and *rsaL* and activates the transcription of both of the genes. Meanwhile, RsaL binds to the intergenic region between *lasI* and *rsaL* and represses the transcription of both genes. CspC binds to the 5′UTR of the *rsaL* mRNA and represses its translation. Acetylation of CspC reduces the affinity between CspC and the *rsaL* mRNA, resulting in upregulation of *rsaL*. The presence of itaconate causes deacetylation of CspC, which represses the translation of the *rsaL* mRNA and subsequently activates the Las system. The 5′ and 3′UTRs of the *rsaL* mRNA are shown in green, and the coding region is shown in gray. Ac, acetylation.

During infection by Gram-negative pathogens, aerobic glycolysis is enhanced in macrophages and monocytes upon sensing LPS, leading to succinate accumulation and generation of reactive oxygen species (ROS) (64). The bactericidal ROS also causes damage to host cells and local tissue. In response to oxidative stress and inflammation, macrophages and monocytes generate and release itaconate, an electrophilic α,β-unsaturated carboxylic acid that suppresses glycolysis, production of proinflammatory cytokines, including interleukin 1β (IL-1β) (65) and IL-6 (66), and reactive oxygen species (65, 67). During lung infection with P. aeruginosa, itaconate is released into airways (68, 69). P. aeruginosa isolates from CF patients have been shown to become adapted to utilize itaconate as a carbon source (39). Itaconate represses LPS synthesis while inducing the production of extracellular polysaccharides in P. aeruginosa, which promotes biofilm formation and chronic infection (39). Here, we found that growth with itaconate as the sole carbon source resulted in lower levels of acetylation of CspC in wild-type PA14 than that when glucose was the sole carbon source. By using the CspC K41R and K41Q mutants, we found that deacetylation of CspC might increase the affinity of CspC to the *rsaL* mRNA, resulting in repression of its translation (Fig. 7). RsaL was identified as a negative regulator of the *lasI* gene (70). The *rsaL* gene and the *lasI* gene are located next to each other and transcribed in opposite directions (Fig. 7) (34). A previous study demonstrated that LasR and RsaL can simultaneously bind to the intergenic region between *rsaL* and *lasI* (23, 24). After binding to LasI-generated 3-oxo-C12-HSL, LasR activates the transcription of both *rsaL* and *lasI* (13). On the contrary, RsaL represses the transcription of the two genes (Fig. 7) (23, 24). Besides *lasI*, RsaL has been found to directly repress the transcription of *phzA1*, *phzM*, and *hcnA*, which are involved in the synthesis of pyocyanin and hydrogen cyanide, respectively (23). A chromatin immunoprecipitation sequencing analysis further identified that RsaL binds to the promoter regions of *pqsH* and *cdpR* and activates their expression (25). *pqsH* encodes the monooxygenase that converts HHQ to PQS (15). CdpR is a transcriptional regulator that directly activates the transcription of *pqsH* (25). These results demonstrate a complex role of RsaL in regulating the QS circuits and genes regulated by the QS systems.

We found that mutation of *cspC* in wild-type PA14 and the Δ*rsaL* mutant decreased the mRNA level of *lasR* compared to the corresponding parental strains (Fig. 2C and 4A), indicating that CspC positively regulates *lasR* independent of RsaL. The transcription of *lasR* is directly activated by Vfr independent of cyclic AMP (cAMP) (71, 72). However, cAMP is required for the positive autoregulation of Vfr, thus rendering cAMP indirectly involved in the regulation of *lasR* (71). Further studies are needed to examine whether CspC influences the expression of *vfr* or the adenylate cyclase genes *cyaA* and *cyaB*. Another possibility is that CspC affects the stability of the *lasR* mRNA. Binding of CspC to the *lasR* mRNA might confer protection against RNases. Combining the results from *lasR*-*lacZ* transcriptional and translational fusions might clarify the regulatory mechanism.

Collectively, our results reveal a regulatory pathway in P. aeruginosa that modulates the QS systems in response to host metabolite. In the presence of macrophage-generated itaconate, the acetylation level of CspC is low, which represses the translation of *rsaL*, leading to activation of the Las QS system that promotes biofilm formation. In combination with our previous report, we postulate that acetylation of CspC in P. aeruginosa acts as a switch between acute and chronic infection. Upon entering the host, CspC is acetylated due to temperature upshift, which results in upregulation of *exsA* and subsequent T3SS genes. Meanwhile, RsaL contributes to the homeostasis of the Las QS system. When macrophage-generated itaconate is accumulated at the infection site, CspC is deacetylated, resulting in downregulation of *rsaL* and upregulation of the QS systems, which might promote chronic infection. Further studies are warranted to examine the expression of the T3SS genes in response to itaconate at body temperature and elucidate the mechanism that controls the acetylation of CspC in response to environmental signals.

## MATERIALS AND METHODS

### Bacterial strains and plasmids.

Primers, plasmids, and bacterial strains used in this study are listed in Table S1 in the supplemental material. The detailed methods are provided in Text S1. Bacteria were grown in LB or the M9 minimal medium at 37°C with agitation. For the M9 minimal medium with a sole carbon source, 22.2 mM glucose (C_6_H_12_O_6_) or 26.7 mM itaconic acid (C_5_H_6_O_4_) was used in the medium to achieve the same molar carbon atoms, and the pH of the medium was adjusted to 7.4. Antibiotics were purchased from BBI Life Sciences, Shanghai, China, and used at the following concentrations: tetracycline, 50 μg/ml for P. aeruginosa and 10 μg/ml for E. coli; carbenicillin, 150 μg/mL for P. aeruginosa; and ampicillin, 100 μg/mL for E. coli.

10.1128/mbio.00547-22.3 TEXT S1 Strain and plasmid construction. Download Text S1, DOCX file, 0.02 MB. Copyright © 2022 Li et al. 2022 Li et al. https://creativecommons.org/licenses/by/4.0/ This content is distributed under the terms of the Creative Commons Attribution 4.0 International license.

### Pyocyanin production assay.

The pyocyanin levels were determined as previously described (73). Bacteria were grown in indicated medium at 37°C overnight. The supernatant was collected by centrifugation at 13,000 × *g* for 2 min; 1 mL of the supernatant was mixed with 600 μL chloroform. The lower chloroform layer was mixed with 300 μL 0.2 M HCl, followed by centrifugation at 13,000 × *g* for 5 min. The upper layer was taken for the measurement of OD_520_ by a microplate reader (Bio-Rad, USA).

### RNA isolation and qRT-PCR.

Total bacterial RNA was isolated with a bacterial total RNA kit (Zomanbio, Beijing, China). cDNA synthesis and qRT-PCR were performed as previously described (33). The ribosomal protein gene *rspL* was used as the internal control.

### QS signal molecule reporter assay.

The QS signal molecule reporter assay was performed as previously described, with minor modifications (74). A PAO1 *lasI rhlI* double mutant containing a *lasB*-*gfp* (ASV) or an *rhlA*-*gfp* (ASV) translational fusion was used as the reporter strain to determine the relative levels of 3OC12-HSL or C4-HSL (35). Wild-type PA14, the Δ*cspC* mutant, and the complemented strain were grown in the ABTGC medium [2.2 g/liter glucose, 2 g/liter Casamino Acids, 1.827 g/liter MgCl_2_.6H_2_O 0.1995 g/liter (NH_4_)_2_SO_4_, 0.3816 g/liter Na_2_HPO_4_, 0.2933 g/liter KH_2_PO_4_, 9.981 mg/liter CaCl_2_, 1.4598 mg/liter FeCl_3_, 0.292 mg/liter NaCl] overnight. Supernatants of the bacterial cultures were collected by centrifugation. The reporter strains were grown in ABTGC medium, followed by mixing with the same volume of the collected bacterial supernatant to achieve an OD_600_ of 0.01 in each well of a 96-well plate. The plate was incubated at 37°C, and the green fluorescent protein (GFP) fluorescence and OD_600_ were measured every 30 min for 12 h.

For the E. coli QS signal reporter strains, measurement of the signal molecule 3-oxo-C12-HSL or C4-HSL was performed as described previously (75). The P. aeruginosa strains were grown overnight and the supernatants were collected by centrifugation; 1 mL of the supernatant was mixed with 4 mL DH5α (OD_600_, 0.1) containing pECP64 and a *lasB*-*lacZ* translational fusion or pECP61.5 and an *rhlA*-*lacZ* translational fusion (75). When the OD_600_ reached 0.3, 1 mM isopropyl-β-d-thiogalactopyranoside (IPTG) was added. After 3 h, the β-galactosidase activities were measured as previously described (33).

### Electrophoretic mobility shift assay.

A total of 40 ng ssDNA was incubated with the purified GST-CspC protein at indicated concentrations for 30 min at 16°C as previously described (33). The electrophoresis was performed at 120 V on ice for 1 h on an 8% native polyacrylamide gel that had been prerun for 1 h in Tris-borate-EDTA (TBE). The DNA bands were visualized after staining with the SYBR Gold nucleic acid gel stain (ThermoFisher Scientific).

### Western blot.

Proteins from the same amount of bacteria were separated by SDS-PAGE and then transferred to a PVDF (polyvinylidene difluoride) membrane. The membrane was blocked with 5% nonfat milk in PBS containing 0.1% Tween 20 (PBST) for 1 h at room temperature, followed by probing with antibodies against glutathione-*S*-transferase (GST) (Sigma, USA), RNA polymerase α antibody (BioLegend), or an anti-acetyllysine antibody (Jingjie PTM Biolab, China) at room temperature for 2 h. After washing with PBST four times, the membrane was incubated with corresponding secondary antibodies at room temperature for 2 h. The membrane was washed four times with PBST, and the signals on the membrane were detected with the Immobilon Western kit (Millipore).

### RIP–qRT-PCR.

RIP was performed as previously described (33). The CspC-GST recombinant protein was purified with a GST tag protein purification kit (Beyotime, China) in the presence of 1 mM dithiothreitol and an RNase inhibitor (Beyotime, China). The eluted protein samples were subjected to RNA isolation with a bacterial total RNA kit (Zomanbio, Beijing, China). After cDNA synthesis, qRT-PCR was performed with primers targeting different parts of the *lasI*, *rsaL*, and *exsA* mRNA. The ribosomal protein gene *rspL* was used as the internal control.
