# Supplementary figures and images for: Acetylation of CspC Controls the Las Quorum-Sensing System through Translational Regulation of rsaL in Pseudomonas aeruginosa
Source: mBio. 2022 Apr 25;13(3):e00547-22. doi: 10.1128/mbio.00547-22 (PMC9239060; doi:10.1128/mbio.00547-22)

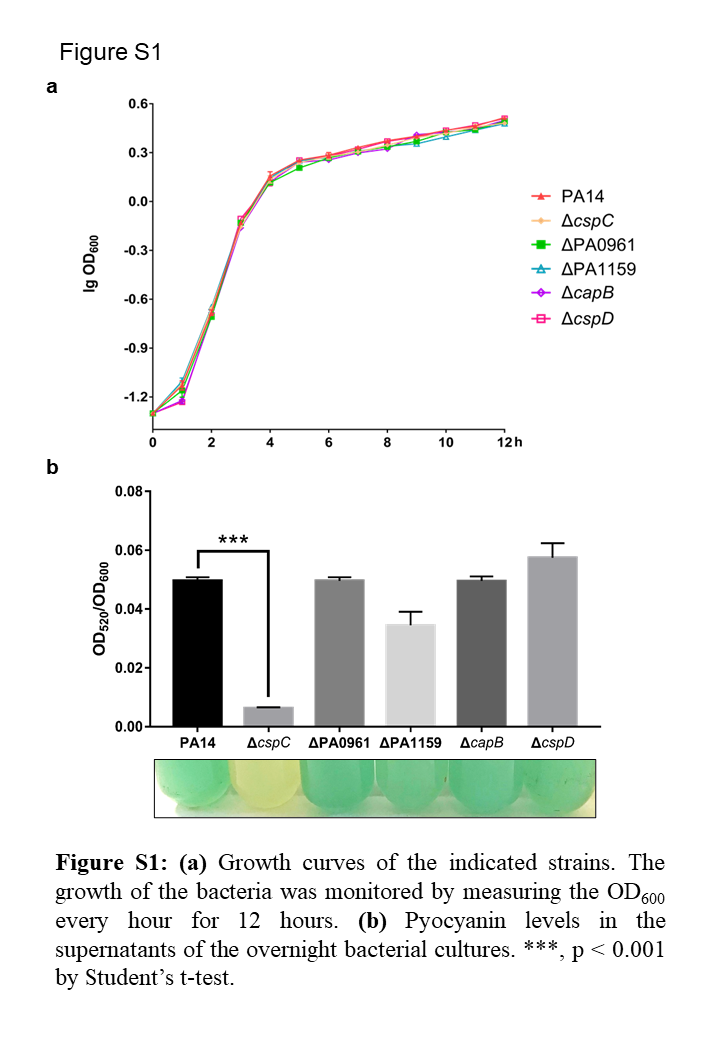

Supplement: FIG S1 [file mbio.00547-22-s0003.tif]
