# Supplementary material for: Acetylation of CspC Controls the Las Quorum-Sensing System through Translational Regulation of rsaL in Pseudomonas aeruginosa
Source: mBio. 2022 Apr 25;13(3):e00547-22. doi: 10.1128/mbio.00547-22 (PMC9239060; doi:10.1128/mbio.00547-22)
Supplement: TABLE S1 [file mbio.00547-22-s0002.rtf]

Table S1. Bacterial strains, plasmids and primers used in this study.
Strain	Description	Source (Reference)	
P. aeruginosa			
PA14	Wild type strain 	(1)	
¦¤cspC	PA14 cspC gene deletion mutant	(2)	
¦¤cspC/cspC	¦¤cspC complementation with cspC inserted on chromosome, Gmr	(2)	
¦¤PA0961	PA14 PA0961 deletion mutant	(2)	
¦¤PA1159	PA14 PA1159 deletion mutant	(2)	
¦¤capB	PA14 capB gene deletion mutant	(2)	
¦¤cspD	PA14 cspD gene deletion mutant	(2)	
¦¤rsaL	PA14 rsaL gene deletion mutant	This study	
¦¤cspC ¦¤rsaL	PA14 cspC rsaL dual-deletion mutant	This study	
PAO1-lasB-gfp	PAO1 containing lasB-gfp (ASV) fusion	(3)	
PAO1-rhlA-gfp	PAO1 containing rhlA-gfp (ASV) fusion	(3)	
E. coli			
DH5á	F-, ö80dlacZÄM15,Ä(lacZYA-argF)U169,deoR,recA1,endA1,
hsdR17(rk-,mk+),phoA, supE44, ë-, thi-1, gyrA96, relA1	TransGen	
S17-1	recA, pro, hsdR, RP4-2-Tc::Mu-Km::Tn7		
BL21(DE3)	F–,ompT,hsdSB (rB–, mB–),gal,dcm(DE3)	Thermo Scientific	
Plasmid			
pEx18Tc-¦¤cspC	cspC gene deletion suicide plasmid	This study	
pEx18Tc-¦¤rsaL	rsaL gene deletion suicide plasmid	This study	
pUC18T-mini-Tn7T-Gm-cspC	pUC18T-mini-Tn7T-Gm with wild type cspC; Gmr	(2)	
pUC18T-mini-Tn7T-Gm-cspC£¨K41Q£©	pUC18T-mini-Tn7T-Gm with cspC K41Q mutant; Gmr	(2)	
pUC18T-mini-Tn7T-Gm-cspC£¨K41R£©	pUC18T-mini-Tn7T-Gm with cspC K41R mutant; Gmr	(2)	
PlasB-lacZ	pDN19lacÙ with lasB promoter; Tcr	This study	
PrhlA-lacZ	pDN19lacÙ with rhlA promoter; Tcr	This study	
PlasI-lacZ	pDN19lacÙ with lasI promoter; Tcr	This study	
PrhlI-lacZ	pDN19lacÙ with rhlI promoter; Tcr	This study	
pUCP20-PcspC-cspC-GST	cspC promoter of PA14 fused to cspC-GST on a promoterless pUCP20; Apr	This study	
pMMB67EH- cspC WT-GST	Wild type CspC-GST driven by an inducible tac promoter; Apr	(2)	
pMMB67EH- cspC K41Q-GST	cspC K41Q-GST driven by an inducible tac promoter; Apr	(2)	
pMMB67EH- cspC K41R-GST	cspC K41R-GST driven by an inducible tac promoter; Apr	(2)	
pMMB67EH- cspC K48Q-GST	cspC K48Q-GST driven by an inducible tac promoter; Apr	(2)	
pMMB67EH- cspC K48R-GST	cspC K48R-GST driven by an inducible tac promoter; Apr	(2)	
pMMB67EH-74-rsaL-GST	74- rsaL -GST driven by an inducible tac promoter; Apr	This study	
pMMB67EH-37-rsaL-GST	37- rsaL -GST driven by an inducible tac promoter; Apr	This study	
pMMB67EH-rsaL-GST	rsaL -GST driven by an inducible tac promoter; Apr	This study	
pGEX-6P-2- cspC WT	pGEX-6P-2 with wild type CspC; Apr	(2)	
pGEX-6P-2- cspC K41Q	pGEX-6P-2 with cspC K41Q mutant; Apr	(2)	
pGEX-6P-2- cspC K41R	pGEX-6P-2 with cspC K41R mutant; Apr	(2)	
Primer	Sequence(5'¡ú3')*	Purpose	
rsaL-Up-F	GGGGTACCTCTATCGCACCACCCA	rsaL deletion	
rsaL -Up-R	CGGGATCCTGCTCTGATCTTTTCGGAC	rsaL deletion	
rsaL -Down-F	CGGGATCCGATCGCCAGCTCGCCG	rsaL deletion	
rsaL -Down-R	CCCAAGCTTGGTATTCAGTTCGCATAAA	rsaL deletion	
Com-PA0456-F	CGGGATCCGCCTGGATCGGCGTCAT	cspC cloning for chromosome insertion	
Com-PA0456-R	CCCAAGCTTACTCTGGGCGGGAACTCG	cspC cloning for chromosome insertion	
Com-0456-GST-R	CCCAAGCTTTTATTTTGGAGGATGGTCGC	cspC cloning with pUCP20	
PA0456-6P-F	CGGGATCCATGTCCCGTCAGAACGGCAC	cspC cloning with pGEX-6P-2	
PA0456-6P-R	CCGCTCGAGGTTGATCACCTGAACGC	CspC cloning with pGEX-6P-2	
PA0456-F	CACACAGGAAACAGAATTCATGTCCCCTATACTAGGTTAT	cspC cloning with pMMB67EH	
PA0456-R	GGTCGACTCTAGAGGATCCTTAGTTGATCACCTGAACGC	cspC cloning with pMMB67EH	
RT-phzA-F	CCACTACATCCATTCCTTC	RT-PCR	
RT-phzA-R	AATTTCTGCATCGGGTTC	RT-PCR	
RT-phzM-F	GACGGCTACGCTAATACC	RT-PCR	
RT-phzM-R	GTAGAACAGCACCATGTC	RT-PCR	
RT-lasI-F	CTGGGCGAGATGCACAAGT	RT-PCR	
RT-lasI-R	AGCAACCGAAAACCTGGG	RT-PCR	
RT-lasR-F	CGAGCGACCTTGGATTC	RT-PCR	
RT-lasR-R	GTAGTTGCCGACGATGAA	RT-PCR	
RT-rhlI-F	CGAATTGCTCTCTGAATC	RT-PCR	
RT-rhlI-R	TTCTCGATGAAGACTTGA	RT-PCR	
RT-rhlR-F	GAAATCGCCATCATCCTG	RT-PCR	
RT-rhlR-R	CGTCGAACTTCTTCTGGA	RT-PCR	
RT-rpsL-F	TATGCCGTGTACGTCTGA	RT-PCR	
RT-rpsL-R	CACTACGCTGTGCTCTTG	RT-PCR	
RIP-21-lasI-F	CTATTTGGAGGAAGTGAAG	RT-PCR	
RIP+68-lasI-R	CACGCAACTTGTGCATCT	RT-PCR	
RIP+433-lasI-F	CCGTAGGGGTGGAGAA	RT-PCR	
RIP+553-lasI-R	GGGTCTTGGCATTGAG	RT-PCR	
RIP-74-rsaL-F	CTAGCAAATGAGATAGATTTCG	RT-PCR	
RIP+36-rsaL-R	CATGTTTTGGGGCTGT	RT-PCR	
RIP+70-rsaL-F	GGGAAAGCCAGGAAAC	RT-PCR	
RIP+178-rsaL-R	AATGCAAAAGCAGGTATA	RT-PCR	
RIP-exsA-74-F	GAAAATCAATAAAACGGAG	RT-PCR	
RIP-exsA-74-R	TATAAGAACCCCAACACTT	RT-PCR	
*, restriction endonuclease recognition sites are underlined.
References
1.	Liberati NT, Urbach JM, Miyata S, Lee DG, Drenkard E, Wu G, Villanueva J, Wei T, Ausubel FM. 2006. An ordered, nonredundant library of Pseudomonas aeruginosa strain PA14 transposon insertion mutants. Proc Natl Acad Sci U S A 103:2833-8.
2.	Li S, Weng Y, Li X, Yue Z, Chai Z, Zhang X, Gong X, Pan X, Jin Y, Bai F, Cheng Z, Wu W. 2021. Acetylation of the CspA family protein CspC controls the type III secretion system through translational regulation of exsA in Pseudomonas aeruginosa. Nucleic Acids Res 49:6756-6770.
3.	Fong J, Yuan M, Jakobsen TH, Mortensen KT, Delos Santos MM, Chua SL, Yang L, Tan CH, Nielsen TE, Givskov M. 2017. Disulfide Bond-Containing Ajoene Analogues As Novel Quorum Sensing Inhibitors of Pseudomonas aeruginosa. J Med Chem 60:215-227.
