# Supplementary material for: Acetylation of CspC Controls the Las Quorum-Sensing System through Translational Regulation of rsaL in Pseudomonas aeruginosa
Source: mBio. 2022 Apr 25;13(3):e00547-22. doi: 10.1128/mbio.00547-22 (PMC9239060; doi:10.1128/mbio.00547-22)
Supplement: TEXT S1 [file mbio.00547-22-s0001.docx]

**Supplemental Methods. Strain and plasmid construction**

Construction of the in-frame deletion mutant in *P. aeruginosa* was performed as described previously (1). To construct the *cspC* deletion mutant, a 959 bp and a 1109 bp fragments upstream and downstream of the *cspC* coding region were amplified by PCR by using PA14 chromosomal DNA as the template with primers cspC-Up-F, cspC-Up-R and cspC-Down-F, cspC-Down-R (Table S1). The PCR products of the *cspC* upstream and downstream fragments were cloned into the EcoRI-KpnI and KpnI-HindIII sites of the plasmid pEX18Tc, respectively. The resultant plasmid was transferred into an *E. coli* conjugation donor strain S17-1, and then transferred to PA14 by conjugation. The PA14 single-crossover mutants (strains with the plasmid integrated into the chromosome) were selected on LB plates with 100 μg/mL tetracycline and 25 μg/mL kanamycin (to kill the S17-1 donor strain). The single-crossover mutants were grown in LB overnight, and then plated on plates with 5% sucrose to select for double-crossover mutants. The correct deletion mutants were screened by PCR with primers cspC-Up-F and cspC-Down-R. For the deletion of *rsaL*, a 992 bp and a 1057 bp fragments upstream and downstream of the *rsaL* coding region were amplified by PCR by using PA14 chromosomal DNA as the template with primers rsaL-Up-F, rsaL-Up-R and rsaL-Down-F, rsaL-Down-R, respectively (Table S1). The PCR products were cloned into KpnI-BamHI and BamHI-HindIII sites of the plasmid pEX18Tc, respectively. Construction of single- and double-crossover mutants as well as screening for *rsaL* deletion mutants were performed as aforementioned.

For the complementation of *cspC* through chromosomal insertion, a fragment containing the promoter region and *cspC* open reading frame was amplified by PCR with primers Com-PA0456-F, Com-PA0456-R (Table S1). The PCR product was cloned into the BamHI-HindIII sites of the plasmid pUC18T-mini-Tn7T-Gm (2). The resultant plasmid was transferred into the Δ*cspC* mutant by conjugation. Selection of strains with the fragment insertion was performed as previously described (2).

To construct the *cspC*-*gst* translation fusion, the coding region of *cspC* was amplified by PCR with primers PA0456-6P-F and PA0456-6P-R (Table S1). The PCR product was cloned into the BamHI-XhoI sites of the plasmid pGEX-6P-2, resulting in pGEX-6P-2-*cspC* WT. To construct the *cspC*-*gst* overexpression strain, The PCR product amplified by PCR with primers PA0456-F and PA0456-R (Table S1) was cloned into the EcoRI-BamHI sites of the plasmid pMMB67EH.

To express the *cspC*-*gst* in *P. aeruginosa*, the fragment containing the promoter region and *cspC* open reading frame was amplified by an overlap PCR. The *cspC* open reading frame with its promoter region was amplified by PCR using PA14 chromosomal DNA as the template with primers Com-PA0456-F, Com-PA0456-R (Table S1). Another PCR was performed by using pGEX-6P-2-*cspC* WT as the template with the primers PA0456-F and PA0456-R (Table S1). The two PCR products were mixed and PCR was performed with primers Com-PA0456-F and Com-0456-GST-R (Table S1). The resultant PCR product was cloned into the BamHI-HindIII sites of the promoterless plasmid pUCP20 (3), resulting in pUCP20-P*_cspC_*-*cspC*-GST.

References

1. Hoang TT, Karkhoff-Schweizer RR, Kutchma AJ, Schweizer HP. 1998. A broad-host-range Flp-FRT recombination system for site-specific excision of chromosomally-located DNA sequences: application for isolation of unmarked Pseudomonas aeruginosa mutants. Gene 212:77-86.

2. Choi KH, Schweizer HP. 2006. mini-Tn7 insertion in bacteria with single attTn7 sites: example Pseudomonas aeruginosa. Nat Protoc 1:153-61.

3. Li M, Long Y, Liu Y, Liu Y, Chen R, Shi J, Zhang L, Jin Y, Yang L, Bai F, Jin S, Cheng Z, Wu W. 2016. HigB of Pseudomonas aeruginosa Enhances Killing of Phagocytes by Up-Regulating the Type III Secretion System in Ciprofloxacin Induced Persister Cells. Front Cell Infect Microbiol 6:125.
